# Application of a validated prognostic plasma protein biomarker test for renal decline in type 2 diabetes to type 1 diabetes: the Fremantle Diabetes Study Phase II

**DOI:** 10.1186/s40842-024-00191-8

**Published:** 2024-10-10

**Authors:** Timothy M. E. Davis, Wendy A. Davis, Scott D. Bringans, James K. C. Lui, Tasha S. C. Lumbantobing, Kirsten E. Peters, Richard J. Lipscombe

**Affiliations:** 1 Medical School, University of Western Australia, Fremantle Hospital, PO Box 480, WA 6959 Fremantle, Australia; 2 Department of Endocrinology and Diabetes, Fiona Stanley and Fremantle Hospitals, Murdoch, WA Australia; 3 Australian Centre for Accelerating Diabetes Innovations, The University of Melbourne, Melbourne, VIC Australia; 4 Proteomics International, Nedlands, WA Australia

**Keywords:** Type 1 diabetes, Chronic kidney disease, Protein biomarkers, Prediction

## Abstract

**Background:**

There are scant data relating to prognostic biomarkers for chronic kidney disease (CKD) complicating type 1 diabetes. The aim of this study was to assess the performance of the plasma protein biomarker-based PromarkerD test developed and validated for predicting renal decline in type 2 diabetes in the context of type 1 diabetes.

**Methods:**

The baseline PromarkerD test score was determined in 91 community-based individuals (mean age 46.2 years, 56.5% males) with confirmed type 1 diabetes recruited to the longitudinal observational Fremantle Diabetes Study Phase II. The performance of the PromarkerD test in predicting the risk of incident CKD (estimated glomerular filtration rate (eGFR) < 60 mL/min/1.73m^2^ in people without CKD at baseline) or an eGFR decline of ≥ 30% over the next four years was determined. The score can range from 0 to 100%, and is categorized as representing low (< 10%), moderate (10% to < 20%) or high (≥ 20%) risk.

**Results:**

The area under the receiver operating characteristic curve was 0.93 (95% confidence interval 0.87–0.99) for the composite renal endpoint, indicating strong predictive accuracy. The positive and negative predictive values at moderate (10% to < 20%) and high (≥ 20%) risk PromarkerD cut-offs were 46.7–50.0% and ≥ 92.0%, respectively.

**Conclusions:**

These preliminary data suggest that PromarkerD is at least as good a prognostic test for renal decline in type 1 as type 2 diabetes.

## Introduction

Around one third of people with diabetes will develop chronic kidney disease (CKD) [1] and diabetes has emerged as the largest single cause of end-stage renal disease in developed and developing countries [2]. CKD can remain asymptomatic for years before diagnosis, but it is strongly associated with cardiovascular disease (CVD) and premature death [3]. Population-based studies have shown that the risk of CKD appears greater in type 1 versus type 2 diabetes across all age strata [4, 5], and there is evidence of relative underutilization of renoprotective therapies in type 1 diabetes [4].

These observations argue for validated tests that reliably identify the risk of progressive renal disease at an early stage in type 1 diabetes and pre-empt preventive management strategies. Unfortunately, most studies of candidate prognostic biomarkers have been conducted in type 2 diabetes [6] and few biomarker tests are available in clinical practice [6, 7]. We have developed a validated plasma protein biomarker-based prognostic test (PromarkerD®, Proteomics International, Perth, Australia) which has excellent performance characteristics for predicting incident CKD and rapid renal decline complicating type 2 diabetes including independent validation in the in the Canagliflozin Cardiovascular Assessment Study (CANVAS) cohort [8–10]. The aim of the present study was to determine whether PromarkerD has similar clinical utility in type 1 diabetes.

## Methods

The PromarkerD test was developed using samples and data from individuals with type 2 diabetes participating Phases I and II of the representative, community-based, longitudinal, observational Fremantle Diabetes Study (FDS) [11]. There were 1,732 participants in Phase II (FDS2) who were recruited from an urban Australian population base of 150,000 between 2008 and 2011 [11]. Of these, 139 (8.0% of the total FDS2 sample) had type 1 diabetes based on clinical criteria and laboratory confirmation including islet autoantibody status and genotyping for monogenic diabetes [12]. All FDS2 participants were invited to detailed face-to-face assessments conducted biennially which comprised comprehensive questionnaires, a physical examination, and fasting biochemical tests performed in a single nationally accredited laboratory [11, 13].

The PromarkerD test was performed using baseline plasma samples. The test algorithm combines the plasma concentrations of three protein biomarkers, apolipoprotein A-IV (ApoA4), CD5 antigen-like (CD5L) and insulin-like growth factor-binding protein 3 (IGFBP3) which, together with the concomitant age, serum HDL-cholesterol concentration and estimated glomerular filtration rate (eGFR) [14], generate an estimate of the risk of incident CKD (eGFR < 60 mL/min/1.73m^2^ in people without CKD at baseline) or an eGFR decline of ≥ 30% over the next four years [8–10]. An enzyme-linked immunosorbent assay was used to measure baseline concentrations of the three biomarkers, as previously described [15]. PromarkerD scores are predicted probabilities of renal outcomes ranging from 0 to 100% and can be categorized as low (< 10%), moderate (10% to < 20%) or high (≥ 20%) risk as determined by pre-specified cut-offs for optimal sensitivity and specificity [8]. Performance was assessed using the area under the receiver operating characteristic curve (ROC AUC). Additionally, sensitivity, specificity, positive predictive value (PPV), and negative predictive value (NPV) were calculated.

## Results

Of the 139 FDS2 participants with type 1 diabetes, 92 (66%) had renal function assessed at the four-year review. Nine of these eligible participants (9.8%) had incident CKD or an eGFR decline ≥ 30%. The baseline clinical and demographic characteristics of the participants categorized by renal outcome are summarized in Table 1. The baseline eGFR and urinary albumin:creatinine ratio differed significantly between the two groups, and the use of antihypertensive medications by those with renal outcomes was double in those without. In bivariable analysis, plasma concentrations of the biomarkers ApoA4 and CD5L were significantly elevated in the people with prespecified renal outcomes while those of IGFBP3 showed no significant difference. PromarkerD scores were substantially higher in those with incident renal outcomes.

**Table 1:** Bivariable analysis showing differences between those with no incident CKD or eGFR decline ≥ 30% over 4 years and those with incident CKD or eGFR decline ≥ 30% over four years

Variables at baseline	No incident CKD or eGFR decline ≥ 30% over 4 years	Incident CKD or eGFR decline ≥ 30% over 4 years	*P*-value
Number (%)	83 (90.2)	9 (9.8)	
Age at FDS entry (years)	45.3 ± 16.4	54.3 ± 13.5	0.117
Sex (% male)	59.0	33.0	0.170
Age at diabetes diagnosis (years)	23.9 ± 12.3	24.7 ± 12.3	0.860
Diabetes duration (years)	20.0 [9.9–31.3]	31.0 [22.5–37.0]	0.056
Body mass index (kg/m^2^)	25.6 ± 4.3	31.0 ± 7.6	0.066
Systolic blood pressure (mmHg)	137 ± 23	147 ± 17	0.202
Diastolic blood pressure (mmHg)	78 ± 12	76 ± 12	0.704
HbA_1c_ (%)	7.8 [7.2–8.8]	7.8 [6.9–8.2]	0.737
HbA_1c_ (mmol/mol)	62 [55–73]	62 [52–66]	0.737
eGFR (mL/min/1.73m^2^)	97.8 ± 21.4	54.8 ± 20.9	< 0.001
eGFR (%)			< 0.001
≥ 90 mL/min/1.73m^2^	73.5	0.0	
60–89 mL/min/1.73m^2^	18.1	55.6	
45–59 mL/min/1.73m^2^	3.6	11.1	
< 45 mL/min/1.73m^2^	4.8	33.3	
Total serum cholesterol (mmol/L)	4.6 ± 1.0	5.0 ± 0.8	0.324
Serum triglycerides (mmol/L)	1.0 (0.6–1.6)	1.0 (0.7–1.7)	0.629
Serum HDL-cholesterol (mmol/L)	1.59 ± 0.49	1.78 ± 0.47	0.261
Urinary albumin:creatinine ratio (mg/mmol)	1.6 (0.7–3.7)	14.7 (1.7–126.3)	0.014
Antihypertensive medication (%)	32.5	77.8	0.012
Renin-angiotensin system blocking drugs (%)	31.3	66.7	0.060
Lipid-modifying medication (%)	28.9	44.4	0.447
Proteomic biomarkers (μg/mL)			
APOA4	25.9 (13.2–50.8)	46.6 (26.9–80.9)	0.007
CD5L	3.1 (1.3–7.3)	7.3 (4.6–11.7)	0.004
IGFBP3	2.1 (1.5–3.0)	2.6 (1.3–4.9)	0.155
PromarkerD Scores (%)	0.16 (0.01–2.96)	12.18 (6.02–24.65)	< 0.001

Data are presented as percentages, mean ± standard deviation (SD), geometric mean (SD range), or median [interquartile range]. Two-way comparisons were performed using Fisher’s exact test, Student’s *t*-test or Mann–Whitney U-test as appropriate

**Fig. 1:**
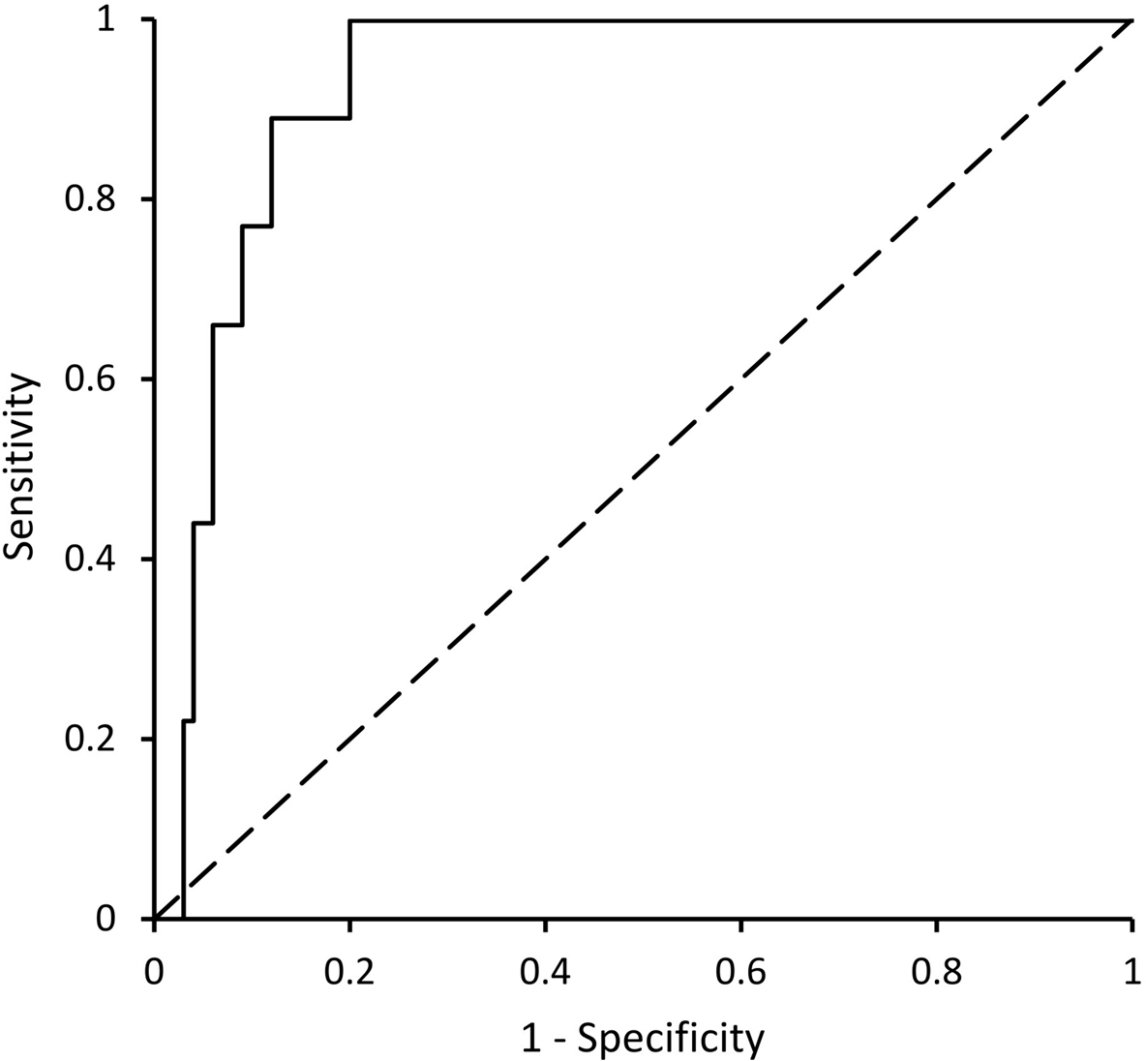
Receiver operating characteristic curve (ROC) for PromarkerD test score as a continuous variable in predicting incident chronic kidney disease or a decline in the estimated glomerular filtration rate of ≥ 30% over four years in individuals with type 1 diabetes

The performance characteristics of PromarkerD are summarized in Table 2. At the moderate risk cut-off, the sum of sensitivity plus specificity was 168.2%, indicating good clinical utility [16]. At the high-risk cut-off, this figure was lower at 119.8% but there were only small number of individuals in this category. At both cut-offs, PPV was moderate (46.7–50.0%) but NPV was very high at ≥ 92.0%. The ROC curve is shown Fig. 1. The ROC AUC was 0.93 (95% confidence interval (CI) 0.87–0.99), consistent with excellent predictive accuracy.

**Table 2:** Predicted versus actual incident CKD or eGFR decline ≥ 30% over four years in individuals with type 1 diabetes and performance metrics at the two PromarkerD risk cut-offs

	No adverse renal outcomes (predicted)	Incident CKD or ≥ 30% 4-year eGFR decline (predicted)	Total (predicted)	Performance metrics			
				Sensitivity (%)	Specificity (%)	PPV (%)	NPV (%)
Moderate risk cut-off (10%)							
No adverse renal outcomes (actual)	75	8	83	77.8	90.4	46.7	97.4
Incident CKD/ ≥ 30% eGFR decline (actual)	2	7	9				
Total (actual)	77	15	92				
High risk cut-off (20%)							
No adverse renal outcomes (actual)	81	2	83	22.2	97.6	50.0	92.0
Incident CKD/ ≥ 30% eGFR decline (actual)	7	2	9				
Total (actual)	88	4	92

## Discussion

The present data provide evidence that PromarkerD has strong clinical utility as a prognostic test of renal decline in type 1 diabetes. The very high ROC AUC of 0.93, and lower 95% CI of 0.87, compare favorably with values of ≤ 0.78 for several panels of protein biomarkers assessed using larger scale samples and data from the Scottish Diabetes Research Network Type 1 Bioresource and Finnish Diabetic Nephropathy Study [17]. The modest PPV at both risk cut-offs needs to be interpreted against the relatively small number of false positive results and the implication that intensive renal risk reduction management, if not needed for CKD, may have other benefits including for CVD risk [18]. The very high NPVs at moderate and high risk cut-offs mean that few people with type 1 diabetes will be burdened by unnecessary management strategies and misplaced concern regarding the future development of CKD.

Although CKD complicating type 1 and type 2 diabetes shares common pathophysiologic determinants, people with type 1 diabetes are generally younger and healthier at diagnosis and carry fewer co-morbidities than those with type 2 diabetes. Consequently, CKD in type 1 diabetes may be less affected by non-glycemic contributing factors including ageing, vascular disease, insulin resistance and obesity [19]. The higher ROC AUC in the present study than in studies of PromarkerD in participants with type 2 diabetes in the FDS2 (0.93 versus ≤ 0.88 [8, 9]), likely reflect this situation, with the PromarkerD biomarker panel less influenced by confounding variables in the present cohort. In addition, the predictive performance of PromarkerD is reassuringly greater than that in a variety of studies in type 1 diabetes, some with smaller sample sizes than the present FDS2 cohort, utilizing available clinical and laboratory data without biomarker concentrations to predict renal decline [20].

The present sample size was constrained by the recruitment strategy for FDS2 and was consequently small since type 1 diabetes constitutes < 10% of all diagnosed diabetes in Australia [12]. We had relatively few pediatric participants but would likely have captured most people in the catchment area with type 1 diabetes given the prominent peaks in incidence in adolescence and middle age [21]. The low numbers precluded analysis of whether PromarkerD had the same performance in sub-groups such as those defined by race or ethnicity. There is a clear need for validation of the present findings in independent cohorts of people with type 1 diabetes, ideally community-based, with sufficient baseline data (demographic and laboratory variables including age, serum HDL-cholesterol and eGFR as well as PromarkerD protein biomarker assays) and subsequent follow-up renal function measurements (over at least four years) to allow the same assessment of PromarkerD prognostic performance. Larger samples would be preferable, but there is the potential to combine the data from other small-scale studies with those of the present study to increase statistical power and to facilitate sub-group analyses. The strengths of the present study include its representative, community based sample [11] and the availability of detailed phenotypic data including rigorous diagnostic criteria for diabetes type [12].

## Conclusion

Although further validation studies are needed, the present data suggest that PromarkerD has strong clinical utility in identifying people with type 1 diabetes at risk of future adverse renal outcomes.

## Data Availability

Data are available from the authors upon reasonable request.
